# A Low-Stress Method for Determining Static and Dynamic Material Parameters for Vibration Isolation with the Use of VMQ Silicone

**DOI:** 10.3390/ma16082960

**Published:** 2023-04-07

**Authors:** Krzysztof Nering, Konrad Nering

**Affiliations:** 1 Faculty of Civil Engineering, Cracow University of Technology, 31-155 Cracow, Poland; 2 Faculty of Mechanical Engineering, Cracow University of Technology, 31-155 Cracow, Poland

**Keywords:** silicone VMQ, Young’s modulus, Poisson ratio, damping, laboratory tests, FEM

## Abstract

Progressive urbanisation causes building users to be affected by increasing amounts of noise and vibrations that come from transportation and other building users. This article presents a test method that can be used to identify quantities of methyl vinyl silicone rubber (VMQ) necessary to carry out solid mechanics finite element method simulations such as Young’s modulus, Poisson ratio, and damping parameters. These parameters are necessary to model the vibration isolation used for protection against noise and vibration. The article uses an original combination of dynamic response spectrum and image processing methods to determine these quantities. The tests were carried out using one machine for the range of normal compressive stresses of 64–255 kPa with cylindrical samples of various shape factors in the range of 1–0.25. The parameters for the simulation of solid mechanics in statics were obtained from image processing based on the deformation of the sample under load; for dynamic solid mechanics, the parameters were obtained from the response spectrum of the tested system. The article shows the possibility of determining the given quantities using the original method of the synthesis of dynamic response and FEM-supported image analysis, which states the article’s novelty. Additionally, limitations and preferred ranges of sample deformation in terms of load stress and shape factor are presented.

## 1. Introduction

Progressing urbanisation and the development of transport infrastructure is an inevitable phenomenon in our civilisation. While this advancement provides many conveniences of shortened travel times to points of interest, it also comes with additional nuisances. Such nuisances are noise and vibrations in residential buildings that come from other multifamily buildings, subway, or tram crossings. Stimuli, such as noise and vibration, have a negative impact on the well-being of residents [1,2,3,4,5,6] and, in extreme cases, on their health [7,8,9,10]. First, the risks associated with exposure to these stimuli are sleep disorders [11,12,13], problems with the cardiovascular system [14,15], decreased concentration [16], and general irritability [17,18,19].

Engineering addresses these challenges and proposes various technological and, above all, material solutions to reduce exposure to the above-mentioned stimuli [20]. A very popular solution in the current housing construction is a floating floor [21,22,23]. Its application, thanks to the use of elastic and damping materials, allows for reducing the transmission of impact sounds through the ceilings of the building and, if properly designed, the perception of transport vibrations in the building. It is also worth mentioning the use of elastic materials for joints between structural elements [24,25]. This allows for not only adjusting the dynamic response of the entire structure to seismic excitations [26,27,28] but also reducing the propagation of high-frequency vibrations radiated in the building as noise [29,30,31]. On the other hand, transport vibrations from rail vehicles can be reduced at the source. It requires an appropriate fastening system, e.g., block support, to be used. Such a system reduces vibration above its resonant frequency. Appropriately selected for a specified location, it allows for a significant reduction in the impact that vibrations cause on people inside buildings [32,33,34,35,36,37].

Modelling such solutions is relatively problematic. There are methods that allow the estimation of airborne [38] and impact noise [39] transmission reduction for floating floors. However, these methods are simplified and do not take into account the possible increase in low-frequency noise and changes in the perception of vibrations by building users [40,41,42]. In the case of flexible joints, the reduction in vibration transmission, including those responsible for noise, can be estimated at the structural node [43,44]. In the case of rail fastening systems, there are various methods for estimating the resonant frequency and reducing vibrations in the above resonance zone [45,46,47,48].

The best method to evaluate a given vibroacoustic protection solution is a laboratory test [49,50,51,52]. However, it is time-consuming and expensive; therefore, it is worth looking for solutions using, for example, FEM numerical methods [53,54]. An important problem in FEM methods is the need to introduce the required material data to the model before starting the simulation [55,56]. One can rely on the manufacturer’s data, but in the case of introducing unusual materials, such data may be limited or not suitable for the way the material works (e.g., working of the material in low stress). This problem is particularly important in work focused on the use of elastic materials with nonlinear characteristics. Numerous vibration-isolating materials, such as polymers, are characterised by nonlinearity, which significantly hinders the setting of parameters for the model [57,58,59,60]. Moreover, there are methods that indirectly measure material parameters, e.g., Young’s modulus can be obtained through the evaluation of material hardness. This methodology is also limited in the application of nonlinear materials but can still be successfully used in the material comparison (softer/harder) [61,62,63,64].

This paper proposes a modification and extension of the method of assessing materials according to the standard [65]. The method presented in this paper evaluates static stiffness, dynamic stiffness, apparent Young’s modulus and Young’s modulus, damping parameters, and Poisson ratio. These parameters are necessary for elastic materials for modelling with FEM. The study presented in the article is carried out on one machine. The material for method evaluation was chosen to be methyl vinyl silicone rubber (VMQ). By proposing one extensive procedure, these parameters are obtained. Compared to the existing methods, the method described in this article is a novelty.

## 2. Measurement Procedure

**Figure 1:**
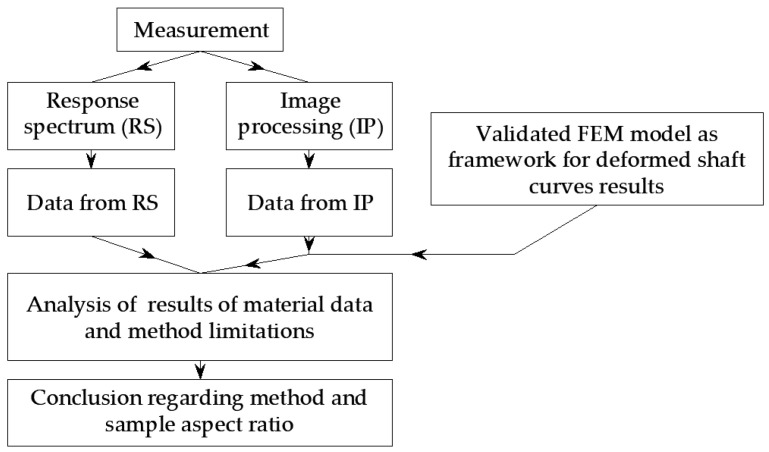
Scheme of research carried out in the article.

The measurements presented in this article were made using a dynamic stiffness testing machine that complies with EN 29052 [65]. However, only the machine itself, i.e., the static load and dynamic excitation method, was used according to the norm. The research carried out in this article includes the simultaneous measurement of dynamic quantities using a response spectrum (RS) and the measurement of volumetric distortions using image processing (IP). The diagram of the measurements carried out is shown in Figure 1.

The purpose of the measurements is to estimate the material parameters by the two above-mentioned methods using RS and IP. Subsequently, the results obtained from these methods are compared. The list of measured parameters is presented in Table 1.

Measurements were taken using cylindrical samples. Each sample differentiated by radius and height had measurements taken in four settings, i.e., 4, 8, 12, and 16 pieces. The dimensions of the samples are shown in Table 2. The upper limit of a sample height was 20 mm. This limit resulted from the fact that the higher samples were subject to sway buckling, which made it impossible to perform the tests.

**Figure 2:**
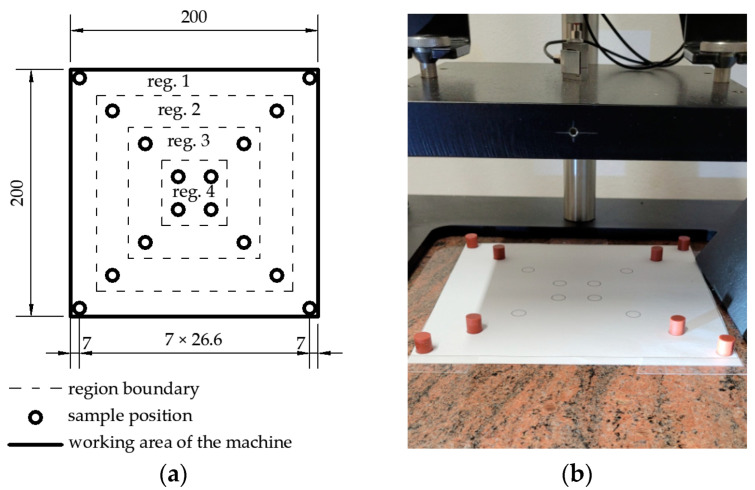
Arrangement of samples during the test: (**a**) plan of the arrangement of samples on the machine plate with regions in which samples were placed sequentially (dimensions in millimetres); (**b**) photo of the sample testing r = 5 mm, h = 10 mm, 8 pcs (loaded regions 1 and 2).

The samples were placed on a baseplate of the testing machine. The first measurement was performed with samples placed in region 1 (4 pcs of samples). Then samples in region 1 were left, and samples in region 2 were added (8 pcs of samples). The procedure was repeated for the following regions: region 1, 2, 3 (12 pcs of samples) and region 1, 2, 3, 4 (16 pcs of samples). In order to find appropriate confidence intervals for the measured quantities, the tests were repeated in 6 sets of samples. The tested system and a photo of an exemplary measuring system are shown in Figure 2.

Such a bisymmetrical system allowed the piston-like operation of the measurement system in each of the 4 settings. This allowed the use of a physical mass model based on a damper and a spring (single degree of freedom). Moreover, the tested samples were subjected to different pressures in order to find possible nonlinearities of material characteristics. Table 3 presents the set of regions loaded during the test and the corresponding regions.

### 2.1. Response Spectrum

The response spectrum was taken using a dynamic stiffness testing machine in accordance with EN 29052 [65]. This machine is generally used to determine the dynamic stiffness (MN/m^3^) parameter for continuous vibration isolation. This parameter allows for predicting a decrease in the impact sound level, which is crucial to ensure protection against impact noise in buildings at the design stage. The work [66] shows the possibility of testing the critical damping factor using the half-power bandwidth method. As part of these studies, a pseudo-displacement spectrum obtained from the measured acceleration values was used.

**Figure 3:**
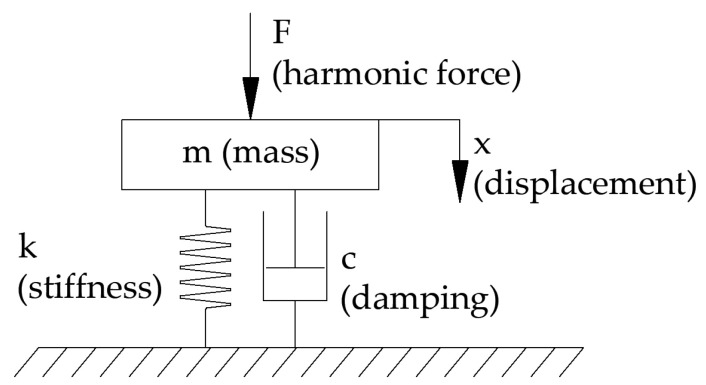
Physical model of the mass–damper–spring system with one degree of freedom.

The machine used for the tests in this article can be approximated by a physical model in the form of a mass–spring–damper with one degree of freedom (SDOF). The harmonic force F in the range of 19–100 Hz is applied to the system using a dynamic exciter (Brüel & Kjær Mini-shaker Type 4810) in the form of a frequency sweep. The lower limit of 20 Hz is related to the resonance of the whole machine, as it is not possible to measure vibroacoustic quantities below that frequency reliably. The upper limit is set for practical reasons since all measured sample systems had a resonant frequency below 100 Hz. The amplitude of the harmonic force was 1 N; therefore, the initial pressure on the plate was 1.5 N so as not to detach the exciter piston with its negative displacement. The pressure plate, with dimensions of 200 × 200 mm, along with the sensors, had a mass of m = 8176.5 g. The stiffness k and damping c of the system are measured based on the resonant frequency and the quality factor (damping, in fact) of the system. The physical model is shown in Figure 3. It can be compared with the Kelvin–Voigt material model. In this study, only frequency response is considered using this model. The rheological behaviour is beyond the scope of this paper.

Measurement of system response in the form of accelerations was recorded using an IEPE accelerometer-MMF KS78B.100 placed on a pressure plate. Control force measurement was performed using a Force sensor—Forsentek FSSM 50 N placed between an exciter piston and a pressure plate.

Before proceeding to record the vibration accelerations, samples were loaded with a pressure plate to which a force was applied and to which vibration accelerations were recorded. The duration of this initial load with the plate and the initial pressure of the exciter was 30 min. This time was necessary to reduce the impact of short time rheological effects in the tested samples. Then, using an exciter, a harmonic force was applied to the sample in the form of a frequency sweep with a frequency increment of 0.1 Hz per 10 s.

**Figure 4:**
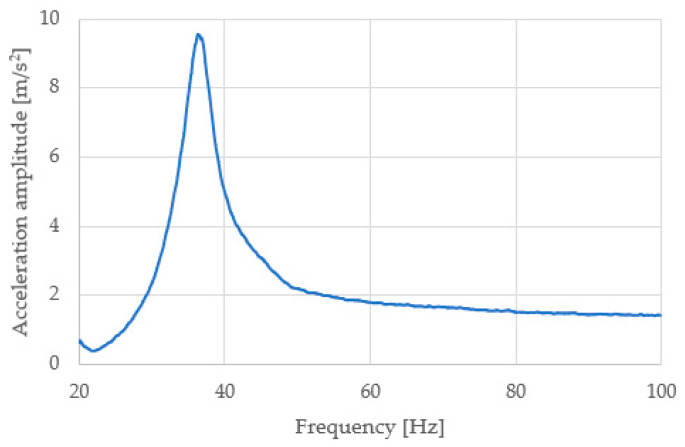
Acceleration amplitude spectrum (blue line) for the sample set *h* = 10 mm and 8 pcs. The resonant frequency of the tested system is equal to 36.4 Hz.

The result of the measurement carried out on the machine used for these tests is the amplitude spectrum of the accelerations. This spectrum allows us to indicate the resonant frequency of the system using the mass–spring–damper model. In the case of low damping, finding the resonant frequency is to indicate the ordinate for the maximum value of the amplitude spectrum, as shown in Figure 4.

It is assumed that the motion is fully harmonic and the initial values of velocities and accelerations equal 0, which means that the pressure plate is at rest before the test, and only the harmonic force sets it in motion. This assumption is needed to directly calculate the pseudo-displacement spectrum, which is necessary to estimate the attenuation using the half-power bandwidth method. The term “pseudo” comes from the fact that when integrating the vibration acceleration signal, it is assumed that the integration constants are equal to the above-mentioned [67]. Therefore, to make the acceleration transition between the spectrum and the spectrum of pseudo-displacements, it is enough to use Equation (1) [67]:

(1) $$\left|X(f)\right|=\frac{\left|a(f)\right|}{{\left(2\pi f\right)}^{2}}$$

where *X(f)* is the pseudo-displacement spectrum, *a(f)* is the acceleration spectrum, and *f* is the frequency.

The half-power bandwidth method allows for determining the critical damping factor. There are two steps to receiving the result. The first step is to estimate the critical damping factor *δ* according to Equation (2) [66,68,69]

(2) $$\frac{f_{2}-f_{1}}{f_{r}}=\frac{\delta }{\pi \sqrt{1-{\left(\frac{\delta }{2\pi }\right)}^{2}}}$$

**Figure 5:**
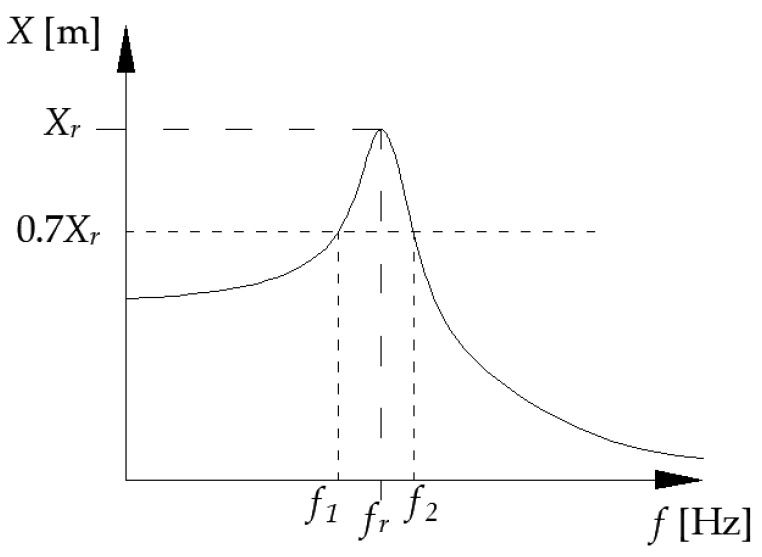
Schematic presentation of the half-power bandwidth method using the displacement spectrum. *X_r_*—displacement amplitude, *f_r_*—resonant frequency, *f*_1_ and *f*_2_—corresponding frequencies to 0.7 value of resonance amplitude.

The presentation of the variables used in Equation (2) is shown in Figure 5.

The second step is to calculate the critical damping factor *D* based on the relationship given in Equation (3) [66,70].

(3) $$\delta =\frac{2\pi D}{\sqrt{1-D^{2}}}$$

### 2.2. Image Processing

The purpose of performing a measurement using image processing is to directly investigate the static deflection and Poisson’s ratio. Both quantities are obtained from the image on its analysis. The measurement height is given by the number of pixels that define the pattern of the sample. This measurement takes place before and after compression, which allows the static deflection to be determined.

The measurement of the Poisson ratio is a bit more complex. The general rule is to measure the shape of the sample sidewall before and after loading. Based on this shape and its dimensions, the Poisson ratio is determined.

There are scientific studies that allow for relating the shape of a cylindrical sample’s sidewall after compression with the Poisson ratio [71,72,73,74,75,76]. There are two states that are distinguished: Simple compression (no friction at the bases of the cylinder) and bonded compression (infinite friction at the bases of the cylinder). These works raise the issue of the shape of the sidewall for flat cylindrical samples (shape factor > 1). After compressing the sample, the effect of this is that the side surface has a parabolic shape [71,72,73,74,75] or a half-sine shape [76]. For samples with a higher height (shape factor < 1), this is not consistent with observations where the shape of the sidewall was observed closer to the polynomial form. However, the conclusion from the above scientific works is that the shape of the sidewall after compression of the sample is correlated with the vertical strain and the Poisson factor.

This article assumes that the samples are subject to bonded compression. The bases of the cylindrical samples were cleaned with isopropyl alcohol before the test in order to ensure the highest coefficient of friction.

**Figure 6:**
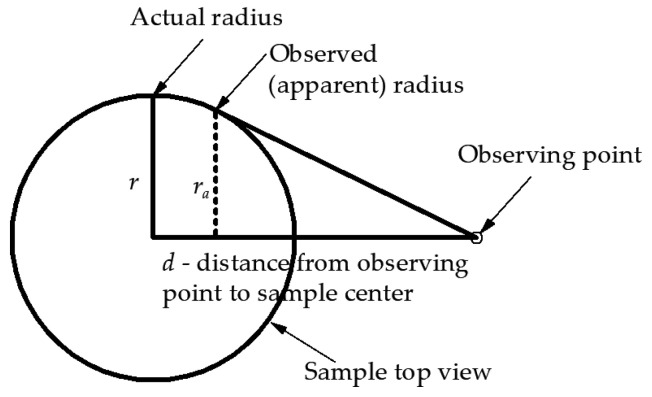
Schematic drawing (top view) of sample sidewall visibility problem.

The images were recorded under controlled conditions. The camera was placed on a tripod and levelled, and it was in one position throughout the study. In order to determine the size of the radius of the sample at a certain level of the tested cylinder (sample), the image was recorded from a distance of about 1.1 m; this minimises the error of the observed sample diameter relative to the actual diameter (Figure 6). In addition, to reduce the error in width measurement, a matrix in the camera with a horizontal dimension 2.5 times larger than the diameter of the sample was used.

Based on the diagram presented in Figure 6, a formula can be postulated describing the relative error of the radius seen in the cylinder sidewall (Equation (4)).

(4) $$\frac{r_{a}}{r}=\sqrt{1-{\left(\frac{r}{d}\right)}^{2}}$$

Based on Equation (4) in Table 4, the necessary distance of the observing point from the centre of the sample is shown in order to obtain the *r_a_/r* ratio data.

**Figure 7:**
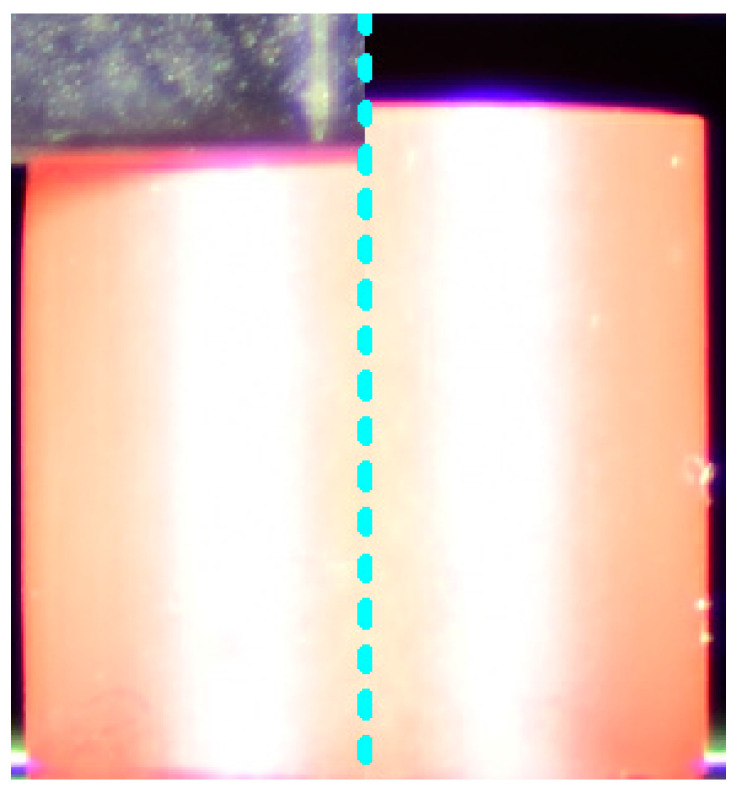
Compressed sample (**left** side), uncompressed sample (**right** side). Sample dimensions *r* = 5 mm; *h* = 10 mm; sample quantity during test: 4.

An example image before being analysed is presented in Figure 7.

**Figure 8:**
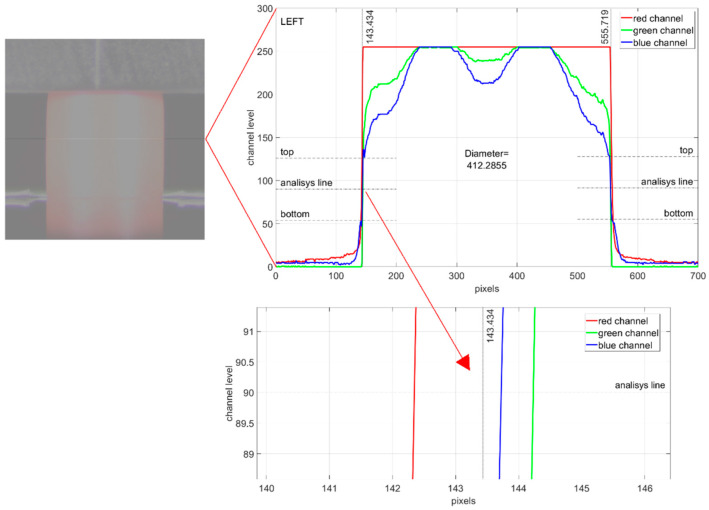
Boundary analysis of the specimen using RGB decomposition. Red arrow indicates analisys line region (zoomed).

Analysis of the sample dimensions was carried out using a simple algorithm implemented in the MATLAB programme. The consecutive lines of the image (1 image pixel high) in the area where the sample was located were analysed. Then, for each line, the image was decomposed into R (red), G (green), and B (blue) channels, which can be seen in Figure 8. The RGB levels ranged from 0 to 255. The area of the greatest growth was analysed separately for the left and right edges’ colour level for all colour components (limiting lines: “top” and “bottom”). Then, for the line located in the middle between the lines limiting the analysis area (“analysis line”), the coordinates of the intersection of the “analysis line” with the lines describing the colour levels were determined. The coordinates of the sample edge were determined as the averages of the intersection points of the individual RGB channel-level graphs with the “analysis line”.

### 2.3. FEM Model

Due to the fact that there is no simple way to estimate the Poisson ratio under bonded compression conditions, FEM simulations were performed to determine the dependence of the sidewall shape on the Poisson coefficient and vertical strain.

The models for each dimension of the samples were made in the FEM simulation software Comsol Multiphysics. The variable parameters for the model were the vertical strain and Poisson ratio. Vertical strain ranged from 0 to 10% in 0.1% increments. The Poisson ratio was established in the range of 0.300 to 0.499 in steps of 0.001. This resulted in a total of 20,200 combinations of vertical strain/Poisson ratio for each of the sample shape factors tested.

The boundary condition for the bottom of the sample was to block the shift in each direction (infinite friction and a rigid substrate). For the top of the sample, blocking horizontal shifts (infinite friction) and forcing in the form of uniform vertical strain. There was also the possibility of providing friction at the top and bottom of the modelled cylinder. This could provide nonlinearity to the model and increase the computational cost significantly. An alternative method would be to provide continuous spring support in the horizontal direction (tangent in both directions to the cylinder bases). This computationally inexpensive method has a huge drawback of an unknown spring stiffness/real friction relationship. Nevertheless, the surface between the machine and the samples was cleaned using isopropyl alcohol to ensure the assumed boundary conditions.

**Figure 9:**
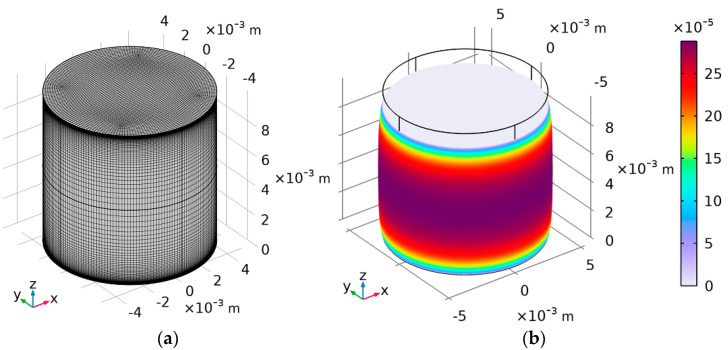
(**a**) FEM mesh for one of the models *r/h* = 0.5 (*r* = 5 mm, *h* = 10 mm). (**b**) Result for von Mises stresses with deformation *e_z_* = 10%, *ν* = 0.48.

The mesh for FEM analysis was built with hexahedra elements with quadratic formulation and reduced integration. Since there is a large change in the horizontal strain near the base of the cylinder, the mesh had to be thickened. The change in the horizontal strain, however, does not occur significantly near the centre of the height of the cylinder, so it was possible to allow the reduction in finite elements to optimise the model. Therefore, an exponential growth rate was set starting from the base of the cylinder with a total number of elements along the sidewall of 10 times the height of the sample. Exemplary mesh and results for 10% vertical strain with Poisson equal to 0.48 are shown in Figure 9.

**Figure 10:**
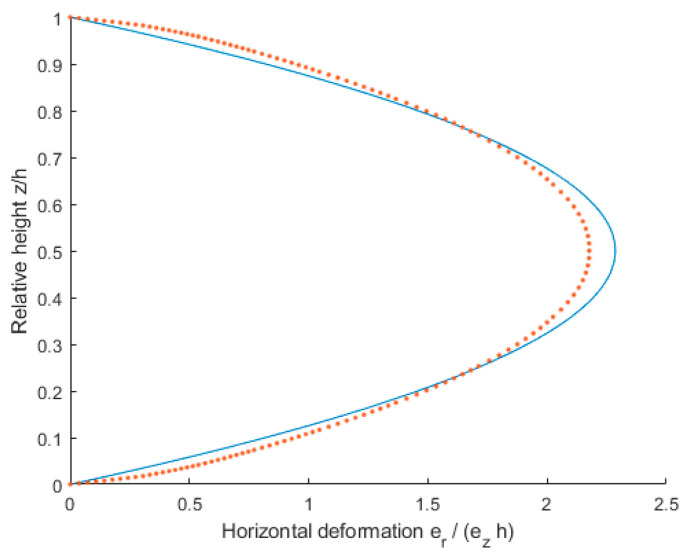
Comparison of the results obtained from [76] and the model made for the research presented in the article. Input parameters *e_z_* = 5%, *ν* = 0.49, *r/h* = 5, error RMSE = 0.1110. Continuous line—result from [76]; dotted line—result from FEM model.

For the parameters of the FEM models made for the purposes of the study, validation was performed using results from the literature [76]. The results obtained from the previously mentioned work for *e_z_* = 5%, *ν* = 0.49, *r/h* = 5 were selected for validation. A comparison of the results is shown in Figure 10.

The error RMSE = 0.1110 can be considered low enough. Furthermore, the shape of the sidewall after compression of the sample is correct despite some underestimation at half height. The model can be considered validated.

### 2.4. Bulge Shape Fitting–Lowest RMSE Fit

**Figure 11:**
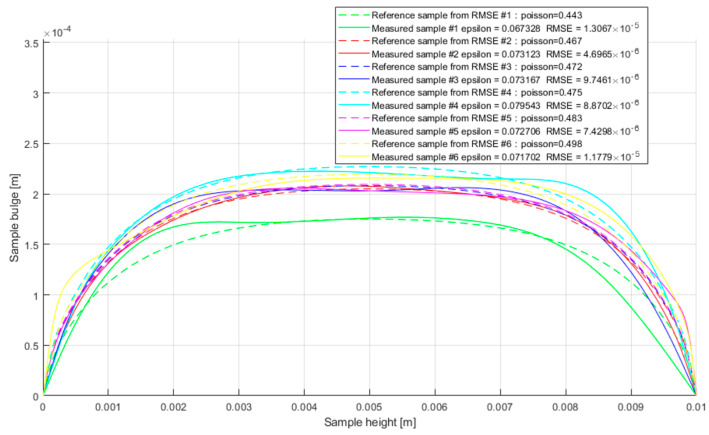
Set of results for Bulge shape fitting using lowest RMSE fit criterion.

In order to obtain the Poisson’s ratio, all curves obtained by image processing were compared with the curves obtained from the FEM model mentioned earlier. This comparison aimed to find the best fitting curve from a model with a given Poisson ratio to these registered during image analysis. The exemplary results obtained during the experiment are presented in Figure 11.

## 3. Results

This chapter presents the results obtained from the measurements according to the procedure described previously.

### 3.1. Response Spectrum

Table 5 presents the results of the measurements of the quantities described in Section 2. The results include the resonant frequency given in the unit of Hertz with the corresponding confidence interval of 95% and the critical damping factor in the dimensionless unit presented analogously.

**Figure 12:**
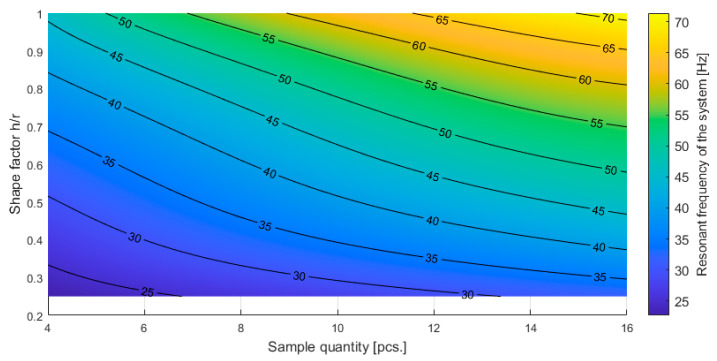
Resonant frequency of the tested system as a function of the quantity of the sample and shape factor of individual samples.

**Figure 13:**
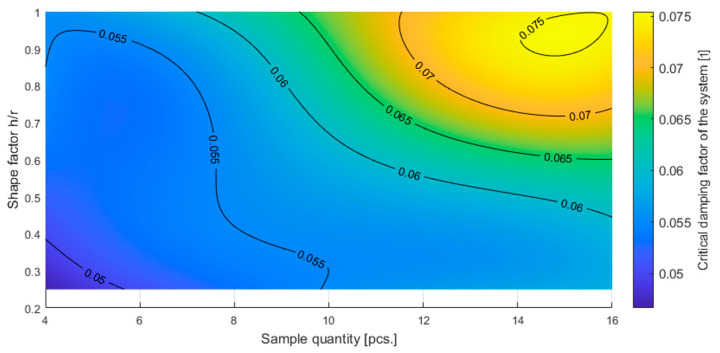
Critical damping factor of the tested system as a function of the quantity of the sample and shape factor of individual samples.

The results presented in Table 5 can be presented graphically, showing the dependence of the measured quantities on the shape factor and sample quantity as in Figure 12 and Figure 13.

### 3.2. Image Analysis

Below in Table 6 are presented results obtained from image analysis.

## 4. Discussion

The research carried out in this article allows us to determine certain derived quantities based on the results obtained.

### 4.1. Derived Quantities

#### 4.1.1. Rayleigh Damping

Rayleigh damping is widely used in modelling larger structures such as buildings [77,78,79]. It is also used for smaller objects [79]. Rayleigh damping does not require the application of nonlinearities in the model by default and allows for changing the damping along with the frequency of the modelled system. It also allows multimodal, wide-spectrum frequency response prediction [80]. It is described by the formula below.

(5) $$D=\frac{1}{2}\left(\frac{\alpha }{\omega_{n}}+\beta \omega_{n}\right)$$

where *D* is the damping factor [-], α is the mass matrix modifier, *β* is the stiffness matrix modifier, and *ω_n_* is the natural angular frequency of the system.

**Figure 14:**
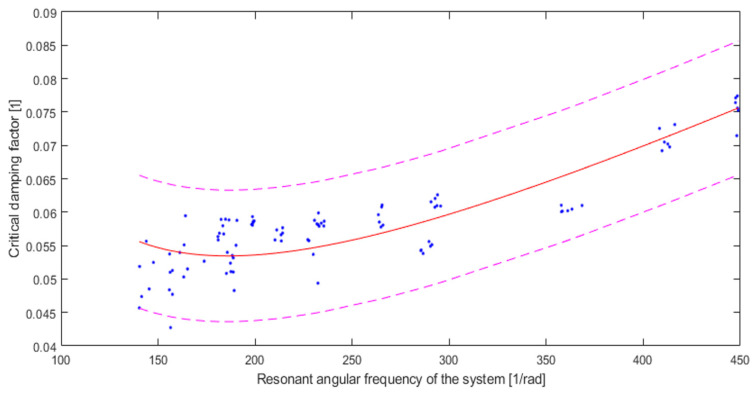
Fitting for Rayleigh damping (solid red line) of measured values (dots) with simultaneous observation confidence bounds 95% (dashed line). R-square: 0.6911.

The results obtained from the critical damping factor tests were adjusted to the Rayleigh damping. The results of this fit are shown in Figure 14.

In Table 7, the results of fitting the curve corresponding to Rayleigh damping to the measurement results are shown.

The fit results show an R-square around 0.7, which may indicate a limited possibility of predicting results using Rayleigh damping parameters. However, it can be concluded that the tested system shows the possibility of modelling its behaviour using Rayleigh damping. However, modelling such systems can be limited only to those in which damping is not the most crucial element.

#### 4.1.2. Spring Stiffness, Static and Dynamic—*k_stat_* and *k_dyn_*

Static stiffness can be determined for the system directly from static deflection. On the basis of the static deflection, the value of the spring stiffness *k* can be determined directly from the definition.

(6) F=kx

where *x* is static defection. By transforming this formula for the purposes of the article, the dependence is obtained.

(7) $$\sigma_{z}\pi r^{2}=ke_{z}h$$

*σ_z_* is the vertical strain, r is the radius of the sample base, *e_z_* is the vertical strain of the sample, and h is the height of the sample. Hence, the apparent Young’s modulus *E_a_* can be determined from Hook’s law.

(8) $$\sigma_{z}=E_{a}e_{z}$$

The apparent Young’s modulus *E_a_* is determined assuming that there are no radial displacements in the compressed sample (the material has the Poisson coefficient *ν* = 0). The assumption is not true because the tested material belongs to the group of incompressible materials (*ν* ≈ 0.5), and the difference between the apparent Young’s modulus and Young’s modulus will be significant for stocky samples (with a high shape factor) [71].

Dynamic stiffness can be determined in another way. By using the mass–spring–damper model, the resonant frequency, the static deflection of the sample group, and the apparent Young’s modulus can be estimated. Static deflection is found from the dependency Equation (9):

(9) $$f_{r}=\frac{1}{2\pi }\sqrt{\frac{g}{e_{zd}h}}$$

where *g* is the acceleration due to gravity (9.81 m/s^2^), *e_zd_* is the vertical strain of the sample (dynamic), and *h* is the height of the sample. Static deflection is the product of vertical strain and height of the sample. Here, however, it should be noted that the vertical strain (*e_zd_*) calculated in this way will correspond to dynamic stiffness, not static stiffness.

**Figure 15:**
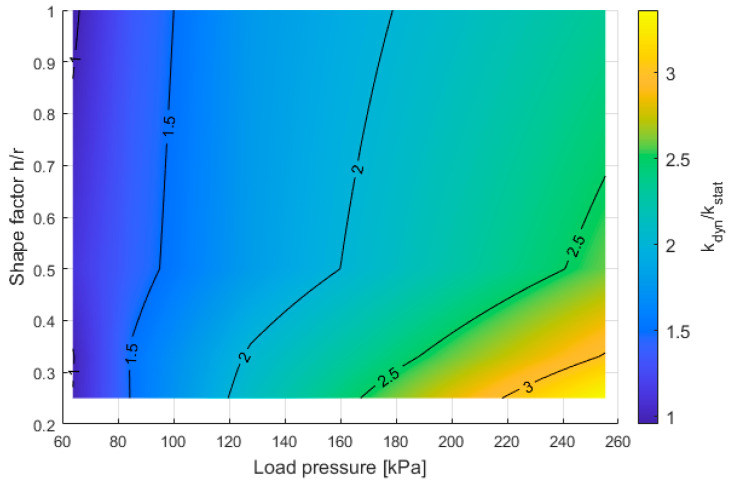
Relationship between the load pressure on the sample with its shape factor and *k_dyn_*/*k_stat_*.

The comparison of static stiffness and dynamic stiffness in the form of *k_dyn_*/*k_stat_* depending on the shape factor and load pressure on the sample is presented in Figure 15.

The above results indicate that at very low loads (around 60 kPa) and thus low deformation of the material, the results for dynamic stiffness and static stiffness are the same for each shape factor—the *k_dyn_*/*k_stat_* is equal to 1. An increase in *k_dyn_*/*k_stat_* can be observed with increasing sample load. As the load increases, the shape factor becomes significant, where at a stress of 250 kPa, depending on the shape factor, *k_dyn_*/*k_stat_* is in the range of 2.2 to over 3.0. Of course, it is worth mentioning that the large increase in load causes the appearance of dynamic stiffening, where *k_dyn_*/*k_stat_* for shape factor 0.25 varies from 1 to more than 3 in the range of 70–250 kPa.

#### 4.1.3. Damping Coefficient

From the results, one can also obtain the damping coefficient. The damping coefficient is a variable used directly in the mass–damper–spring system—similar to the stiffness *k*. The method of obtaining the damping coefficient *c* is described by the following relationship:

(10) $$D=\frac{c}{c_{c}}$$

(11) $$c_{c}=2\sqrt{mk}$$

where *c* is the damping coefficient, *c_c_* is the critical damping coefficient, and *m* is the damped mass in the system.

**Figure 16:**
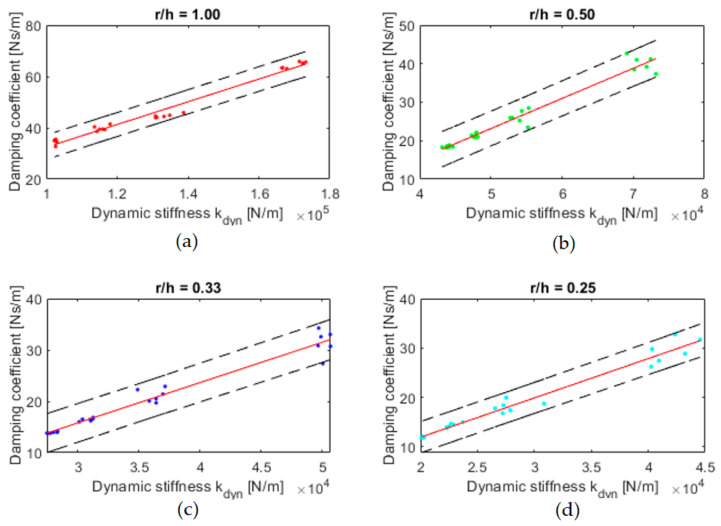
Presentation of linear fitting (function *a x* + *b*) for dynamic stiffness and damping coefficient: (**a**) *r/h* = 1.00 (red dot), (**b**) *r/h* = 0.50 (green dot), (**c**) *r/h* = 0.33 (blue dot), (**d**) *r/h* = 0.25 (cyan dot). Red lines are considered as best fitting curve and black dashed lines are confidence interval limits. Coefficients of linear fitting and R-square are presented in Table 8.

Taking into account the fact that the samples are arranged in one system, a parallel system, the results of the above quantities can be presented for a single sample. As can be seen in Figure 16, it is possible to determine the dynamic stiffness and damping coefficient for a single sample for each shape factor.

As a supplement to Figure 16, Table 8 shows coefficients of linear fit for dynamic stiffness and damping coefficient relationship.

The R-square results are highly satisfactory, and they are above 0.95. An interesting observation is that the angle of inclination of the curve that determines the dynamic stiffness and damping coefficient relationship is similar for shape factor 0.25–0.50, while for 1.00, it is lower. This may indicate the maximum damping capacity of the tested material when the shape factor increases.

#### 4.1.4. Loss Factor

By using the Kelvin–Voigt material model, the loss factor can be estimated. From the results obtained for the critical damping factor, by using Formulas (12) and (13) [81,82], the phase shift between stress and strain can be calculated.

(12) $$\tan\left(\delta \right)=\frac{E\prime \prime }{E^{\prime }}=\eta$$

(13) η=2D

where *E″* is loss modulus, *E′* is storage modulus, and *δ* is loss angle (phase shift between stress and strain in material).

Mathematically, a loss factor is similar to a critical damping factor. It often happens that FEM software accepts a loss factor instead of a critical damping factor. Therefore, for convenience, these results are shown in Table 9.

Considering a structure that is entirely bench-like, Rayleigh damping is the simplest to implement because it is damping related to the system geometry. In the case of using a different structure but using the tested material in a similar arrangement, the damping coefficient *c* is preferred, which can be set as the boundary condition of the structural support. The most general way to approach a material is through the critical damping factor/loss factor. Figure 3 basically presents the Kelvin–Voigt model for the site studied. On this basis, the attenuation parameters present in the article were calculated. A loss factor (4.1.4) has also been added to this article as an important consideration of damping in the Kelvin model.

### 4.2. Poisson Ratio

**Figure 17:**
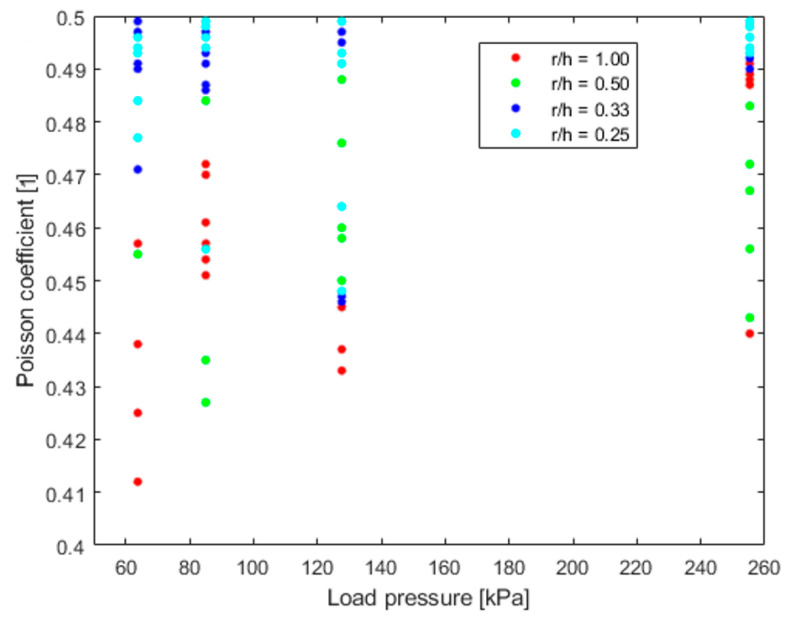
Poisson ratio obtained from z lowest RMSE curve fitting for each load and sample.

The results presented in Table 6 allow for concluding that the Poisson ratio for the tested material is in the range of 0.46–0.49. Initially, it can be seen that the results depend on the shape factor or the load. However, for a more accurate evaluation of the measurement method, it is worth seeing the results for individual measurements. A graphical summary of these results is shown in Figure 17.

By analysing the above results in the context of the shape factor, it can be said that for *r/h* = 1.00 and 0.50, the results show higher discrepancies. This translates into the accuracy of the recorded results and the length over which they are recorded. With shorter sidewalls, the maximum bulge values are over a shorter distance. The lengthening of the bulge registration section by lengthening the sample has a positive effect on the result. A longer side wall in samples with *r/h* = 0.33 and 0.25 accompanies the results with a smaller discrepancy. It has a similar effect on the divergence of load pressure results. Here, however, the higher the load pressure, the better the convergence of the results, in all cases, shape factor. This is influenced by a smaller error when registering larger deformations using image processing. This relationship can be seen in the summary of the above results in Table 10.

### 4.3. Poisson Ratio Using Apparent Young’s Modulus

An alternative approach to calculating the Poisson ratio can also be used. By knowing the apparent values of the Young’s modulus, an analogous assumption can be made as in the case of the calculation of the Young’s modulus; thus, *Ea/E* = 1.03 for an *r/h* = 0.25 (assumption for high Poisson ratio). Then, by using Formula (14), the Poisson ratio can be estimated based on the *E_a_* values calculated from the measurements for each shape factor.

(14) $$\frac{E_{a}}{E}=\frac{1+3\nu \left(\frac{1-\nu }{1+\nu }\right)S^{2}}{1+3\nu \left(1-2\nu \right)S^{2}}$$

By using the above formula as a curve-fitting model, the Poisson ratio results shown in Table 11 were obtained. Moreover, by knowing that k_dyn_/k_stat_ for low load pressures also equals 1, one can check whether the calculated Poisson ratio for dynamic Young’s modulus would be correct.

By using the curve fitting to the model presented by Formula (14), where the parameter is the Poisson ratio, the results converge with those previously observed for static loads. However, there is no valid result for dynamic load. Based on the research conducted in this article, the reason for this observation cannot be clearly identified.

### 4.4. Young’s Modulus

**Figure 18:**
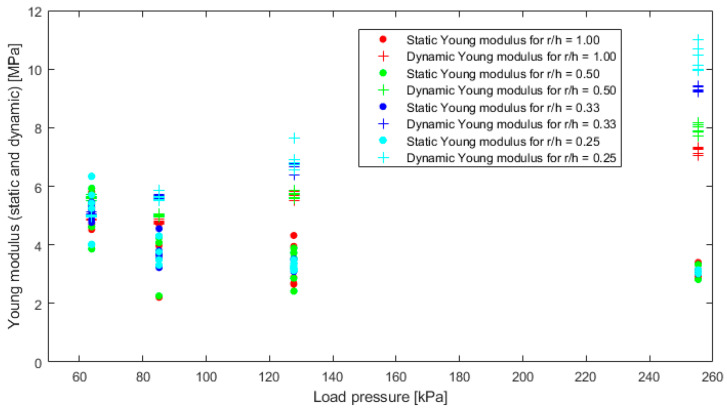
Comparison of static and dynamic Young’s modulus with assumption *E_a_/E* = 1.03 for *r/h* = 0.25.

Based on Formula (8), the apparent Young’s modulus of the material for each load pressure. By knowing the Poisson ratio and using the relationship (14) with the assumption *E_a_/E* = 1.03 for *r/h* = 0.25, Young’s modulus can be determined. The results of this activity are presented in the graph Figure 18.

In the case of calculating the static Young’s modulus, a reduction in the dispersion of the results can be observed with increasing load pressure. However, an interesting observation is a fact that the increase in static load pressure in the dynamic Young’s modulus shows the grouping of the results into individual shape factors.

## 5. Conclusions

The conducted experiment shows the possibility of determining all the quantities necessary to carry out FEM dynamic and static simulations with the use of VMQ silicone, namely, Young’s modulus/dynamic Young’s modulus, damping, and Poisson ratio. The novel methodology presented in this paper is able to support the FEM modelling of vibroisolating structures. A synthesis of response spectrum and image processing provides the necessary support for obtaining material data in a low-stress region, showing material nonlinearity. Nevertheless, the presented methodology has its own limitations.

The Poisson ratio is recorded at the smallest discrepancy in highly deformed samples (with the highest load pressure). Moreover, the length of the deformed sidewall also has a significant impact on the results. The smallest dispersion of results is observed with a slender sample, where *r/h* = 0.25. However, it should be emphasised that the samples in the presented study should not undergo sway buckling. Samples with too high slenderness (low shape factor), in this particular case *r/h* = 0.16, were not suitable to be tested.

It has been shown that it is possible to estimate the Poisson’s ratio not only using curve fitting, especially for low shape factors, but also using Formula (14), given the apparent Young’s modulus for high and low shape factors with static loads applied to samples.

A correlation between the damping coefficient and the dynamic stiffness has been demonstrated. The damping coefficient depends on the degree of loading of the sample, dynamic stiffness, and its shape factor. As the shape factor increases, the damping coefficient increases. Similarly, as the load and the dynamic stiffness increase, the damping coefficient increases.

For the tested VMQ silicone material, for low stresses, the dynamic stiffness/Young’s modulus can be set equal to the static one. The problem, however, appears at higher stresses, where not only does the dynamic stiffening of the material occur due to the increase in load, but also the shape factor becomes an important variable. Therefore, before conducting the simulation, it is recommended to perform the above research procedure to determine the static and dynamic values set in the model.

## Figures and Tables

**Table 1:** Measured parameters as part of methods using response spectrum and image processing.

Response Spectrum		Image Processing	
Directly Measured	Derived Quantities	Directly Measured	Derived Quantities
Resonant frequency [Hz] Acceleration amplitude spectrum [m/s^2^]	Dynamic stiffness [N/m] Critical damping factor [1] Damping coefficient [Ns/m] Loss factor [1] Apparent Young’s modulus (dynamic) [Pa] Young’s modulus (dynamic) [Pa]	Static deflection [m] Sample shaft deformation [m]	Poisson ratio [1] Static stiffness [N/m] Apparent Young’s modulus (static) [Pa] Young’s modulus (static) [Pa]

**Table 2:** Dimensions of the tested samples.

Radius [mm]	Height [mm]	Aspect Ratio of the Cylinder	Sample Unit Weight [g] with 95% CI
5	5	1.0	0.449 (0.448; 0.450)
	10	0.5	0.889 (0.888; 0.901)
	15	0.3333	1.349 (1.346; 1.352)
	20	0.25	1.800 (1.798; 1.801)

**Table 3:** Set of regions loaded and sample quantity with pressure applied to a single sample.

Regions Loaded	Sample Quantity for Single Test	Stress in Single Sample [kPa]
1	4	255
1, 2	8	128
1, 2, 3	12	85
1, 2, 3, 4	16	64

**Table 4:** Determination of the required distance from the sample axis to ensure a given ratio of the apparent radius to the real radius for the sample radius of 5 mm.

*r_a_/r*	*d* [mm]
0.9	11
0.99	35
0.999	112
0.9999	354
0.99999	1118
0.999999	3536

**Table 5:** Results of measurements using the frequency response spectrum method.

Sample Height [mm]	Aspect Ratio of the Cylinder	Sample Quantity	Resonant Frequency [Hz] CI 95%	Critical Damping Factor [1] CI 95%
5	1.00	4	45.9 (45.6; 46.1)	0.0554 (0.0548; 0.0559)
		8	57.7 (57.2; 58.1)	0.0616 (0.0601; 0.0630)
		12	65.6 (65.1; 66.1)	0.0699 (0.0689; 0.0709)
		16	71.4 (71.4; 71.5)	0.0766 (0.0745; 0.0787)
10	0.50	4	29.5 (29.3; 29.7)	0.0524 (0.0505; 0.0542)
		8	36.6 (35.9; 37.3)	0.0544 (0.0517; 0.0570)
		12	42.1 (41.9; 42.4)	0.0602 (0.0559; 0.0644)
		16	46.6 (46.0; 47.3)	0.0615 (0.0609; 0.0621)
15	0.33	4	24.9 (24.5; 25.3)	0.0473 (0.0456; 0.0489)
		8	30.1 (29.9; 30.3)	0.0574 (0.0529; 0.0620)
		12	33.9 (33.7; 34.1)	0.0553 (0.0542; 0.0564)
		16	37.5 (37.3; 37.6)	0.0583 (0.0579; 0.0587)
20	0.25	4	22.8 (22.4; 23.2)	0.0477 (0.0455; 0.0499)
		8	25.9 (25.3; 26.5)	0.0547 (0.0503; 0.0591)
		12	29.1 (28.7; 29.4)	0.0563 (0.0536; 0.0591)
		16	31.6 (31.5; 31.7)	0.0585 (0.0579; 0.0591)

**Table 6:** Results of measurements using the image analysis method.

Sample Height [mm]	Aspect Ratio of the Cylinder	Sample Quantity	Vertical deformation e_z_ [%] CI 95%	Horizontal Bulge Deformation in Middle e_r,middle_/h [%] CI 95%	Poisson Ratio ν [1] CI 95%
5	1	4	5.530 (5.177; 5.883)	3.390 (3.058; 3.722)	0.482 (0.460; 0.500)
		8	2.670 (2.126; 3.214)	0.789 (0.526; 1.053)	0.469 (0.434; 0.500)
		12	1.563 (1.062; 2.063)	0.286 (0.203; 0.369)	0.461 (0.452; 0.470)
		16	0.935 (0.845; 1.024)	0.121 (0.104; 0.137)	0.447 (0.417; 0.476)
10	0.5	4	7.293 (6.882; 7.704)	4.086 (3.733; 4.439)	0.470 (0.449; 0.490)
		8	3.338 (2.680; 3.997)	0.908 (0.766; 1.050)	0.463 (0.447; 0.480)
		12	1.972 (1.368; 2.576)	0.371 (0.251; 0.491)	0.474 (0.438; 0.500)
		16	1.278 (1.051; 1.504)	0.192 (0.151; 0.234)	0.491 (0.473; 0.500)
15	0.33	4	8.057 (7.937; 8.177)	4.548 (4.449; 4.647)	0.494 (0.490; 0.497)
		8	3.747 (3.531; 3.963)	1.018 (0.914; 1.121)	0.479 (0.452; 0.500)
		12	2.205 (1.951; 2.458)	0.408 (0.362; 0.454)	0.492 (0.487; 0.498)
		16	1.183 (1.118; 1.248)	0.165 (0.152; 0.179)	0.490 (0.480; 0.500)
20	0.25	4	8.044 (7.880; 8.209)	4.356 (4.276; 4.436)	0.496 (0.493; 0.498)
		8	3.796 (3.627; 3.965)	0.994 (0.941; 1.046)	0.481 (0.460; 0.500)
		12	2.236 (1.956; 2.515)	0.399 (0.336; 0.462)	0.490 (0.472; 0.500)
		16	1.246 (0.993; 1.500)	0.165 (0.133; 0.197)	0.490 (0.482; 0.498)

**Table 7:** Fitting parameters for results in relation to Rayleigh damping.

Coefficients (with 95% Confidence Bounds):	Goodness of fit:
α = 9.938 (9.365, 10.51) β = 2.875 × 10^−4^ (2.773 × 10^−4^, 2.978 × 10^−4^)	SSE: 0.001448
	R-square: 0.6911
	RMSE: 0.003925

**Table 8:** Coefficients of linear fit for function *a x* + *b* with 95% confidence bounds for Figure 16.

r/h	a	b	R-Square
1.00	4.43 × 10^−4^ (4.14 × 10^−4^, 4.72 × 10^−4^)	−11.827 (−15.683, −7.971)	0.9784
0.50	7.83 × 10^−4^ (7.15 × 10^−4^, 8.50 × 10^−4^)	−15.984 (−19.731, −12.237)	0.963
0.33	7.85 × 10^−4^ (7.16 × 10^−4^, 8.53 × 10^−4^)	−7.740 (−10.308, −5.171)	0.9621
0.25	7.97 × 10^−4^ (7.38 × 10^−4^, 8.55 × 10^−4^)	−4.007 (−5.721, −2.293)	0.9734

**Table 9:** Results of a loss factor in the tested samples.

Sample Height [mm]	Aspect Ratio of the Cylinder	Sample Quantity	Loss Factor [1] CI 95%
5	1.00	4	0.1107 (0.1097; 0.1118)
		8	0.1231 (0.1202; 0.1261)
		12	0.1398 (0.1378; 0.1418)
		16	0.1532 (0.1489; 0.1574)
10	0.50	4	0.1048 (0.1010; 0.1085)
		8	0.1087 (0.1034; 0.1140)
		12	0.1203 (0.1117; 0.1289)
		16	0.1230 (0.1217; 0.1243)
15	0.33	4	0.0945 (0.0912; 0.0979)
		8	0.1149 (0.1058; 0.1240)
		12	0.1106 (0.1084; 0.1127)
		16	0.1166 (0.1159; 0.1173)
20	0.25	4	0.0955 (0.0910; 0.0999)
		8	0.1094 (0.1006; 0.1181)
		12	0.1127 (0.1071; 0.1182)
		16	0.1170 (0.1158; 0.1183)

**Table 10:** Comparison of average Poisson ratios in different shape factors (1–0.25) obtained from the minimum RMSE curve fit and its standard deviations.

	1	0.5	0.33	0.25
Average Poisson ratio	0.46458	0.47454	0.48875	0.48913
Standard deviation	0.02643	0.02372	0.01427	0.01385

**Table 11:** Results of Poisson ratio with and without assumed for *r/h* = 0.25 *E_a_/E* = 1.03. Analogously for dynamic loads method was applied.

Data Source	Assumption Type	95% Confidence Bounds
Static load	With assumed *E_a_/E* = 1.03 for *r/h* = 0.25	0.4946 (0.4784, 0.5000)
	Without any assumption for *r/h* = 0.25	0.4809 (0.4619, 0.4998)
Dynamic load (only for lowest load, where *k_dyn_* = *k_stat_*)	With assumed *E_a_/E* = 1.03 for *r/h* = 0.25	0.4448 (0.4145, 0.475)
	Without any assumption for *r/h* = 0.25	0.4250 (0.4070, 0.4431)

## Data Availability

Not applicable.
